# Evaluating the effectiveness of dual dye combination of indocyanine green and carbon nanoparticles with parathyroid hormone test in preserving parathyroid gland during papillary thyroid cancer surgery: a single-center retrospective cohort study

**DOI:** 10.1007/s13304-024-01804-8

**Published:** 2024-03-20

**Authors:** Yuliang Chen, Songze Zhang, Keyu Miao, Jiagen Li

**Affiliations:** Department of Thyroid and Breast Surgery, The Affiliated People’s Hospital of Ningbo University, No. 251, Baizhang East Road, Yinzhou District, Ningbo, 315040 Zhejiang China

**Keywords:** Papillary thyroid cancer, Parathyroid glands, Indocyanine green, Nano-carbon, Parathyroid dipstick

## Abstract

Preserving the integrity of parathyroid glands is crucial in papillary thyroid cancer (PTC) surgery to avoid hypoparathyroidism. In recent years, two novel dyes, activated carbon nanoparticles (CNP) and indocyanine green (ICG), have been utilized to assist in parathyroid gland identification. However, the use of CNP or ICG alone can result in extravasation of dye or excessive fluorescence of non-parathyroid tissue, which can affect the accuracy of surgical outcomes by yielding false negative or false positive results. Therefore, it is important to further optimize the application of these two dyes in surgery. We analyzed case files of 124 PTC patients who underwent routine total or near-total thyroidectomy with bilateral lymph node dissection in the central region at the Affiliated People's Hospital of Ningbo University from January to November 2022. The patients were randomly divided into three groups based on the type of intraoperative dye used. The CNP group (*n* = 38) received an intra-thyroidal injection of CNP dye. The ICG group (*n* = 42) used the ICG near-infrared fluorescence endoscopy system to show parathyroid fluorescence. The group that received a combined approach of ICG and CNP (*n* = 44) leveraged the advantages of both methods that allow for positive development of ICG and negative development of CNP to identify and preserve the parathyroid gland during operation. The parathyroid hormone detection reagent (PTH test method) was employed to verify the highly suspected parathyroid tissue in all three groups. We analyzed intraoperative data pertaining to intraoperative parathyroid identification, misexcision, number of autotransplantation, and postoperative hypoparathyroidism among the three groups. Compared with the ICG group and the CNP group, the combined group demonstrated more prominent advantages in identifying average the number of lower parathyroid glands (1.93 ± 0.26, *p* = 0.015), reducing the average numberrate of misexcision (0.45 ± 0.5, *p* = 0.004), and reducing the incidence of postoperative temporary hypothyroidism (3/44, *p* = 0.015). The combined use of ICG and CNP dual-dye with PTH test method appears to be more effective in both identifying and protecting parathyroid glands during PTC surgery.

**Supplementary Information:**

The online version contains supplementary material available at 10.1007/s13304-024-01804-8.

## Introduction

Thyroid cancer is currently a common malignant tumor in clinical practice, with papillary carcinoma being the most frequently encountered pathological type. In 2020, there were about 580,000 new cases of papillary thyroid cancer (PTC) worldwide, and according to statistics, it ranks in the top 10 of all cancers [1]. Patients with PTC usually need to be treated by surgery. Intraoperative parathyroid injury is easy to cause hypoparathyroidism, with the incidence of permanent hypoparathyroidism being approximately 0%–3% [2]. Postoperative hypoparathyroidism is the main cause [3]. Therefore, protecting parathyroid gland is crucial in PTC surgery.

Active carbon nanoparticle (CNP) is a new type of dye. After the injection of CNP into the thyroid gland during PTC surgery, the majority of the thyroid gland and surrounding lymph nodes can be stained black in a short time, which can allow for the negative development of the parathyroid gland. However, CNP staining can sometimes contaminate the surgical field around the thyroid gland, causing accidental removal of the parathyroid gland.

Indocyanine green (ICG) fluorescence endoscopic imaging technology is one of the latest breakthroughs in the field of dyes to protect the parathyroid gland during PTC surgery. Intravenous injection of ICG can rapidly bind to plasma lipoproteins, reach the parathyroid gland through the blood circulation, and excite fluorescence in the near infrared wavelength of about 800 nm. It can be captured by near-infrared sensing cameras [4, 5]. However, ICG can be easily disturbed by light, and intravenous injection of ICG produces fluorescence not only on the parathyroid gland, but also on the adjacent tissues with rich blood supply such as thyroid gland and lymph nodes, which can affect the accurate identification of parathyroid gland during surgery.

Given the advantages and limitations of both techniques, this study aims to combine the two dyes and evaluate their positive and negative development effects under endoscopy. In addition, we recognize the research gap in the identification techniques of the living parathyroid glands. Current identification has been heavily dependent on empirical methods with its accuracy influenced by the experience of surgeons. Therefore, it is imperative to develop a more objective and efficient identification method. Literature suggests that parathyroid hormone test method (PTH test method) could be more effective than the empirical judgment [6, 7]. The objective of this study is to investigate the feasibility and effectiveness of ICG and CNP dual dyes combined with PTH test method in protecting parathyroid glands during PTC surgery.

## Materials and methods

### Design

This retrospective cohort study was reported according to the STROBE guidelines [8]. It received approval from the Ethics Committee of the Affiliated People's Hospital of Ningbo University (2022 Research No. 068), and a written informed consent was obtained from the participants for the publication of the results and any accompanying tables/figures.

### Patients

We collected data from the Department of Thyroid Breast Surgery, People’s Hospital of Ningbo University, Ningbo, China, between January 2022 and November 2022. The data was obtained from patients diagnosed with PTC who underwent total or near-total thyroidectomy with bilateral lymph node dissection in the central region.

### Surgical methods

Our surgical approach involved the use of dyes as a routine procedure. Initially, we experimented with nanocarbon staining alone, which yielded fair staining results. Subsequently, we tried ICG dye alone and achieved favorable results. Based on these findings, we used a combination of two dyes in an attempt to generate synergistic effects. The surgical procedures and data collection were performed by the same surgical team. All patients underwent total or near-total thyroidectomy with bilateral central zone lymph node dissection.

In the combined group, after the thyroid tissue was exposed, an injection of CNP dye was administered into the thyroid gland (Chongqing Lemay Pharmaceutical Co., Chongqing, China). To minimize the risk of accidental CNP dye spillage and contamination of adjacent tissue, the injection site was compressed and coagulated using an electric knife (CNP imaging method). ICG (Chinese medicine approval number H20055881, Dandong Yichuang Pharmaceutical Co., Ltd., Liaoning, China), dissolved in sterile water, was administered intravenously. The ptoMedic NIR fluorescence endoscopy system was used to observe parathyroid fluorescence. A thorough visual examination was then conducted on the dorsal thyroid gland to identify any highly suspected parathyroid tissues. Following thyroidectomy and bilateral lymph node dissection in the central region, the same dose of ICG was reinjected. The glands identified preoperatively were re-evaluated using the ICG imaging method. In the CNP group, only the CNP imaging method was used, while in the ICG group, only the ICG imaging method was employed.

**Fig. 1:**
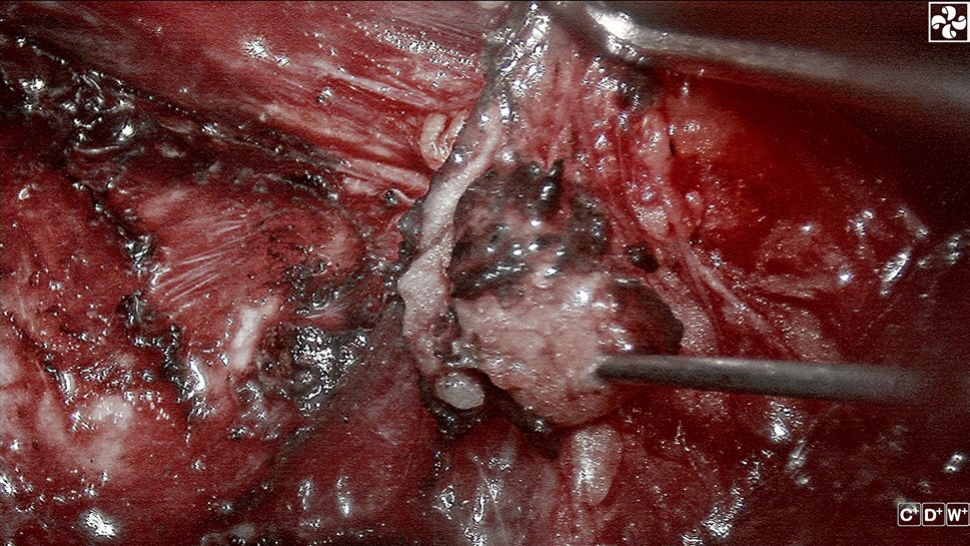
Fine needle aspiration of the parathyroid gland

**Fig. 2:**
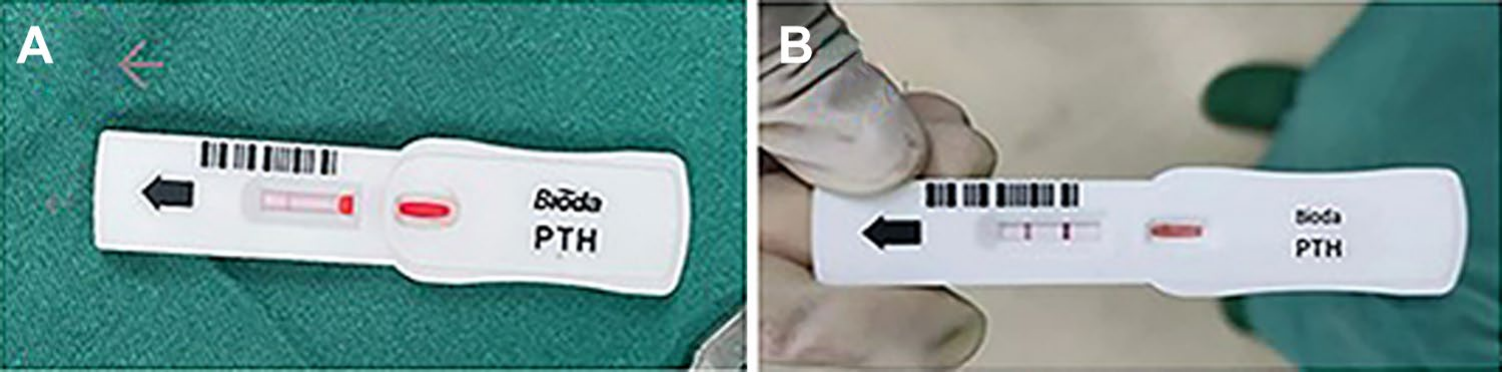
PTH test results shown. Image A indicates that non-parathyroid (PTH test negative), and image **B** indicates that is parathyroid (PTH test positive)

All three groups of patients had highly suspicious parathyroid tissue detected during surgery. For these tissues, those evaluated as having good activity are selected to be retained in situ. We perform fine needle aspiration (Fig. 1). The syringe is combined with a disposable intravenous indwelling needle, and an appropriate amount of the extract in the needle cavity is dripped into the PTH test paper monitoring point. (Eshzhu Zhun 20152402195, Wuhan Baioda Biotechnology Co., Ltd., Wuhan, China), and then the observation time was about 10 min to evaluate the results (PTH test method, Fig. 2). For highly suspicious parathyroid tissue that was mistakenly resected or whose activity was poorly evaluated, we removed 1 mm^3^ tissue samples for frozen section examination. If the parathyroid tissue was identified as parathyroid, autologous transplantation into muscle was performed.

**Fig. 3:**
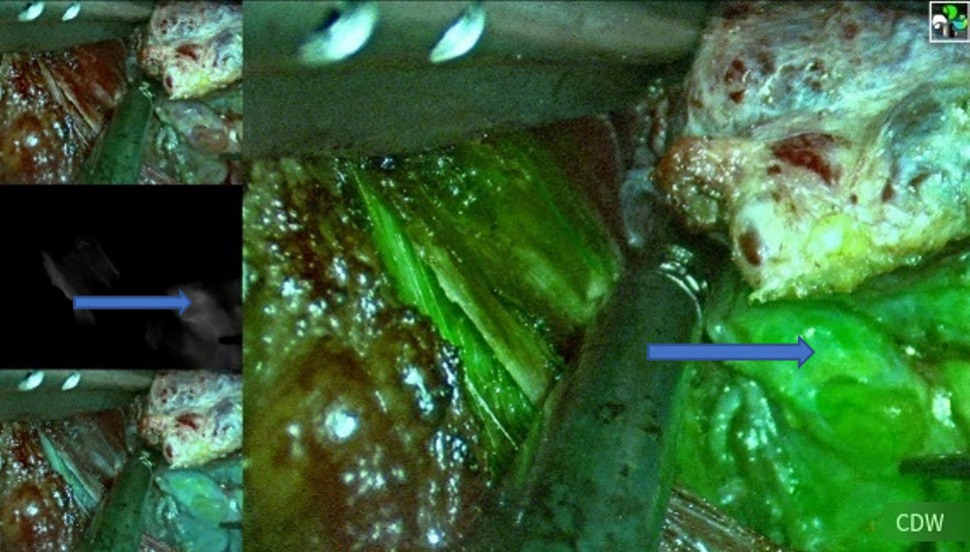
Visualization of a parathyroid gland with a score of 2(blue arrows)1

During surgeries involving ICG, we routinely invited three experienced specialists from outside the team to provide intraoperative support. The fluorescence intensity of parathyroid tissue in both ICG-only and combined groups was assessed using the grading system proposed by Fortuny et al. [9]. An ICG score of 0 indicated no fluorescence was observed, while a score of 1 indicated fluorescence in both the parathyroid gland and its surrounding tissue. A score of 2 (Fig. 3) indicated a higher fluorescence intensity in the parathyroid gland compared to the surrounding tissue (fluorescence grading method) [10]. For cases with a fluorescence grading score of 0, autologous transplantation was performed. If the score was 1, the evaluation was repeated, and if the score remained 1 after re-evaluation, fine needle puncture is performed to observe the blood supply. In cases where parathyroid flow was abundant, the parathyroid glands were preserved in situ, while in cases with no blood flow, autograft transplantation was performed. If the score was 2, in situ preservation treatment was conducted.

To further evaluate the predictive value of fluorescence intensity for postoperative hypoparathyroidism, fluorescence score data were collected from the ICG group and the combined group. Based on the fluorescence intensity results, patients were divided into three groups: group 0, group 1, and group 2. Grouping criteria were as follows: patients with the highest intraoperative parathyroid score of 0 were included in group 0, those with a score of 1 were included in group 1, and those with a score of 2 were included in group 2.

### Data collection

We collected retrospective data collection EMRs (inpatient and outpatient records). These data included information on patients' gender, age, tumor size, height, weight, length of hospital stay, intraoperative operative time, intraoperative bleeding, number of intraoperatively identified parathyroid glands, number of transplanted parathyroid glands, postoperative drainage, and postoperative blood calcium and PTH levels on the first day, the third day, and the first, third, and sixth month after operation.

Postoperative hypoparathyroidism and hypocalcemia were determined based on postoperative PTH levels and blood calcium levels. Temporary hypoparathyroidism was defined as PTH levels < 15 pg/ml within 1 day after surgery or the presence of symptoms such as numbness or tingling in fingertips or perioral, hand, foot, or facial muscles, convulsions or epileptiform generalized convulsions, or multiple episodes of blood calcium < 2.0 mmol/L and blood phosphorus > 2.0 mmol/L, with recovery within 3 months. Permanent hypoparathyroidism was defined as requiring more than 6 months for recovery [11].

### Basis for grouping

The inclusion criteria were the same for all patients: (1) signed informed consent for surgery; (2) age between 18 and 79 years, without gender or race restrictions; (3) PTC confirmed by routine postoperative pathology; (4) lesions with a maximum diameter < 30 mm.

Exclusion criteria included: (1) abnormal preoperative parathyroid hormone (PTH) levels; (2) lesions invading the thyroid peritoneum or surrounding tissues, or retrosternal goiter; and (3) combined parathyroid gland disease.

Ultimately, the clinical data of 136 patients who met the requirements were screened. Among the patients who initially met the inclusion criteria, five patients were excluded due to incomplete postoperative PTH reviews following the standard follow-up schedule. Additionally, seven patients were excluded as a result of early or delayed follow-up. As a result, we obtained and analyzed case data from a total of 124 patients.

Patients were divided into three groups based on the dye used during surgery: CNP group (CNP dye only, *n* = 38), ICG group (ICG dye only, *n* = 42), and combined group (combined CNP and ICG double dye, *n* = 44).

### Statistical analysis

SPSS.25.0 statistical software was used for statistical analysis of all data, and the measurement data of each group were expressed as $(\overline{X} \, + \,S)$. The continuous variable differences between groups were analyzed using one-way analysis of variance (ANOVA), and the pairwise comparisons between groups were analyzed using the Student–Newman–Keuls (SNK) method. The differences in categorical variables were analyzed using the chi-square test. Receiver operating characteristic (ROC) curves were also plotted. *P* < 0.05 was considered statistically significant.

## Results

### Clinical data analysis

#### Comparison of clinical data among the three groups

There were 36 males and 88 females, with an average age of 45.22 ± 12.86 years. There were no significant differences in age, gender, tumor size, BMI index, and length of hospital stay among the three groups. The difference in surgical costs between the three groups was statistically significant (*P* < 0.001, Table 1). After further analysis, the highest surgical cost of the three groups of surgeries was about $741. The cost difference was mainly concentrated in the cost of CNP dye, which was about $45, accounting for about 6% of the total surgical cost.

**Table 1:** Comparison of clinical data among the three groups

Group of groups	CNP group *N*1 = 38	ICG group *N*1 = 42	Combined group *N*1 = 44	F/ chi-square	*P*
Age ($$\overline{X} \, \pm \,s$$, years)	43.45 ± 12.88	45.67 ± 14.19	46.32 ± 11.60	0.542	0.583
Gender (male/female)	9/29	17/25	10/34	4.046	0.132
Tumor size ($$\overline{X} \, \pm \,s$$, mm)	6.95 ± 2.90	6.67 ± 3.11	7.07 ± 2.85	0.207	0.813
BMI ($$\overline{X} \, \pm \,s$$, kg/m^2^)	24.16 ± 2.23	23.69 ± 2.48	23.93 ± 2.03	0.442	0.644
Length of hospital stay ($$\overline{X} \, \pm \,s$$, days)	4.45 ± 1.20	4.48 ± 1.13	4.77 ± 1.18	1.004	0.369
Operation fee(*x* ± *s*, $)	705.85 ± 11.67	708.31 ± 14.69	718.11 ± 12.78	10.234	< 0.001

#### Comparison of surgery-related indicators among the three groups

Compared with ICG group and CNP group, the combined group had more advantages in identifying the average number of lower parathyroid glands and reducing the average number of misexcision (*P* < 0.05). There was no significant difference in intraoperative blood loss, operation time, postoperative drainage volume, and the number of upper parathyroid glands identified among the three groups (*P* > 0.05, Table 2).

**Table 2:** Comparison of surgery-related indicators among the three groups

Group of groups	CNP group *N*1 = 38	ICG group *N*1 = 42	Combined group *N*1 = 44	F/chi-square /*H*	*P*
Intraoperative blood loss ($\overline{X} \, \pm \,s$, ml)	6.26 ± 2.53	6.45 ± 2.43	7.41 ± 2.55	2.537	0.083
Operation time ($\overline{X} \, \pm \,s$, min)	133.97 ± 29.91	134.57 ± 25.69	134.61 ± 27.98	0.007	0.993
Postoperative drainage volume ($\overline{X} \, \pm \,s$, ml)	18.95 ± .17	19.10 ± 7.17	19.89 ± 5.436	0.271	0.763
Number of identified upper parathyroid glands (*x* ± *s*, *n*)	1.89 ± 0.31 ^a^	1.83 ± 0.37^a^	1.93 ± 0.26^b^	1.049	0.354
Number of identified lower parathyroid glands (*x* ± *s*, *n*)	1.68 ± 0.47 ^a^	1.76 ± 0.43 ^a^	1.93 ± 0.26 ^b^	4.335	0.015
Number of misexcision parathyroid glands (*x* ± *s*, *n*)	0.76 ± 0.43^a^	0.74 ± 0.45^a^	0.45 ± 0.5^b^	5.818	0.004
Number of autotransplanted parathyroid glands (*x* ± *s*, *n*)	0.45 ± 0.5^a^	0.83 ± 0.91^b^	0.57 ± 0.73^ab^	2.897	0.059

Note: The superscript letters a and b indicate the categories with significant statistical significance after pairwise comparison (*P* < 0.05)

The average operation time of traditional curved neck incision was (129.59 ± 25.84) minutes, and the average operation time of transthoracic breast incision was (148.25 ± 28.30) minutes. The average operation time was statistically significant between the two approaches (*P* < 0.05), which was considered because the incision needed to establish a cavity for the transthoracic breast approach was longer. Therefore, the intraoperative blood loss and postoperative drainage volume of the two surgical methods were not significantly affected, showing no statistical significance (*P* > 0.05, Table 3).

**Table 3:** Comparison of operation-related indicators of different surgical methods

Methods	Traditional curved neck incision *N*1 = 92	Via breast approach *N*2 = 32	F/ chi square /*H*	*P*-value
Intraoperative blood loss ($\overline{X} \, \pm \,s$, ml)	6.53 ± 2.50	6.66 ± 1.84	0.066	0.782
Operation time ($\overline{X} \, \pm \,s$, min)	129.59 ± 25.84	148.25 ± 28.30	11.791	0.001
Postoperative drainage volume ($\overline{X} \, \pm \,s$, ml)	18.75 ± 6.39	21.00 ± 5.61	3.124	0.08

#### Comparison of postoperative indexes among the three groups

Compared with ICG group and CNP group, the combined group was more effective in reducing the incidence of postoperative temporary hypothyroidism (*P* < 0.05). At the same time, there was no statistically significant difference in the incidence of recent complications such as hoarseness and hypocalcemia among the three groups. For the long-term complication of permanent hypoparathyroidism, we added a control group, and there was no statistically significant difference after comparing the four groups. (*P* > 0.05, Table 4).

**Table 4:** Comparison of postoperative complications among the three groups

Group of groups	Control group *N* = 48	CNP group *N*1 = 38	ICG group *N*1 = 42	Combined group *N*1 = 44	F/ chi square /*H*	*P*
Temporary hypoparathyroidism	15^a^	12^a^	10^a^	3^b^	3.046	0.019
Permanent hypoparathyroidism	2	0	0	0	1.755	0.158
Hoarseness of voice	5	4	2	0	1.875	0.136
Hypocalcemia	4	3	1	0	1.678	0.174

Note: The superscript letters a and b indicate the categories with significant statistical significance after pairwise comparison (*P* < 0.05)

### Expert fluorescence grading score analysis

**Fig. 4:**
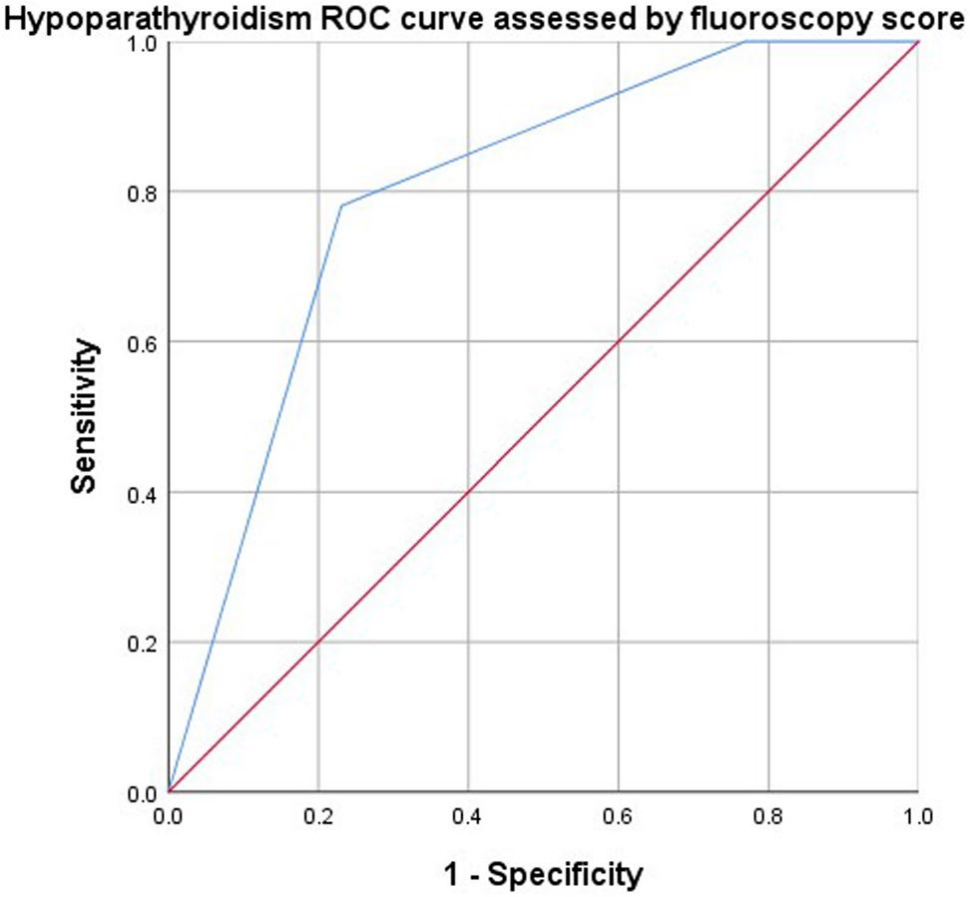
ROC curve for evaluating hypoparathyroidism by fluorescence score

We scored each of the 325 intraoperatively identified parathyroid glands individually using a fluoroscopic grading scale for 86 patients in the ICG and combined groups. The incidence of temporary hypoparathyroidism was significantly lower in group 2 than in score groups 0 and 1 (Table 5), and the area under the ROC curve (AUC) for the diagnostic efficiency of temporary hypoparathyroidism was 0.8, according to the fluorescence grading score. The sensitivity and specificity of the fluorescent parathyroid rating score in predicting post thyroidectomy hypoparathyroidism were 100% and 84.6%, respectively (Fig. 4).

**Table 5:** Postoperative data of patients treated with ICG intravenously

Score of fluorescence	Score 0 group *N*1 = 3	Score 1 group *N*1 = 23	Score 2 group *N*1 = 60	F/ chi square /*H*	*P*
Postoperative temporary hypoparathyroidism (*n*, %)	3^a^ (100%)	7 ^b^ (30.4%)	3 ^c^ (5%)	25.537	< 0.001
Mean calcium level ($\overline{X} \, \pm \,s$, mg/dl)	2.13 ± 0.28	2.49 ± 0.15	2.50 ± 0.17	8.415	< 0.001

Note: The superscript letters a, b, and c indicate the categories with significant statistical significance after pairwise comparison (*P* < 0.05)

## Conclusions

Upon analyzing the perioperative data of 124 patients with PTC, the combined group of 44 patients (1.93 ± 0.26, *p* = 0.015) demonstrated better recognition efficiency in identifying the average number of lower parathyroid glands, compared to the CNP group and ICG group. There were 20 cases of misresection of parathyroid glands in the combined group (0.45 ± 0.5, *p* = 0.004), and the average number of misexcision significantly lower than that of the CNP group and ICG group. In terms of postoperative complications, only three patients in the combined group developed temporary hypoparathyroidism (3/44, *p* = 0.015), which were significantly lower than the CNP group and the ICG group. There were no reported cases of permanent postoperative hypoparathyroidism in any of the three groups. In the analysis of the fluorescence grading scoring method, we found that patients with at least one parathyroid scored 2 (Score 2 group) had a significantly lower incidence of temporary postoperative hypoparathyroidism.

## Discussion

PTC is often treated by surgery. In addition to the protection of the recurrent laryngeal nerve, the identification and preservation of the parathyroid gland are also the key steps of the operation. At present, CNP has been used clinically as a positive dye for lymph nodes. The diameter of CNP is about 150 nm, while the gap between the capillary wall is 30–50 nm, and the gap between the lymphatic capillaries is 500 nm. Therefore, CNP can quickly enter the lymphatic system without entering the blood vessels, which allows for a diffusion of CNP through the thyroid lymphatic system. This process helps the thyroid gland, central region and lateral lymph nodes to be stained without a dyeing on the parathyroid gland. However, using CNP alone may pose a risk of contamination of the surgical field leading to false negative results if extravasation of the drug leaks [12]. Thus, this study combined it with the ICG fluorescence endoscopic imaging technology, which protects the parathyroid gland through intravenous injection of an appropriate dose of ICG, and near-infrared fluorescence imaging.

ICG is an anionic, water-soluble, three-carbon cyanine molecule that rapidly binds to plasma lipoproteins when injected intravenously, reaches the parathyroid gland through the blood circulation, and emits fluorescence once it is excited by the 800 nm near-infrared wavelength. Previous studies show that this molecule can be captured, and it can be developed on, half-life is about 3–5 min, and it can be completely metabolized by the body in about 20 min [13, 14].It can be injected repeatedly. The toxic dose for adults is 5 mg/kg, and the incidence of adverse reactions is about 0.05% [9, 15]. Furthermore, ICG helps distinguish recurrent laryngeal nerve from blood vessels. Although ICG rarely affects the surgical field due to tissue staining caused by leakage, it may visualize tissues with the surrounding rich blood vessels, such as thyroid gland, so it may have a certain impact on the identification of parathyroid gland when used alone.

Furthermore, it is important to note that our method of verifying whether the target gland is indeed a parathyroid gland differs from the conventional approach. The clinical method relies heavily on empirical observations, which can introduce a degree of subjectivity into the process. In vitro tissue, frozen section pathological examination is mostly used, which needs to wait for a long time during the operation. This examination is often limited in some hospitals due to equipment and technical problems. To address this issue, an alternative approach that has been promoted is PTH test method in our center, which uses colloidal gold as the color medium and takes advantage of the principle of specific binding of antigen and antibody in immunology. According to a study by Xia et al., the total coincidence rate between the results of conventional visual detection and intraoperative pathological detection was 74.1% (103/139), and the total coincidence rate between the two was statistically significant (χ^2^ = 41.58, *P* < 0.0001) [7]. However, when using PTH test method, the positive coincidence rate was 97.4% (76/78), and the negative coincidence rate was 100% (61/61), there was no significant difference compared with the intraoperative pathological results (χ^2^ = 2.026, *P* > 0.05). The use of the PTH test method for intraoperative parathyroid identification has been found to be more effective compared to the conventional approach. This method offers several advantages, including convenience, simplicity, and safety. Moreover, it does not have any adverse effects on the function of transplanted parathyroid glands. By employing the PTH test method, surgeons can achieve accurate and reliable identification of parathyroid glands during surgery without compromising their functionality.

In this study, we observed that the combined use of ICG and CNP, along with PTH test method, yielded significant improvements in identifying inferior parathyroid glands, preventing inadvertent dissection of parathyroid glands, and reducing postoperative temporary hypoparathyroidism. Given the retrospective nature of our trial and the fact that the procedures were conducted by a specific team, it is important to acknowledge that the findings may have limited generalizability to other settings and institutions. Further investigation is warranted to assess the applicability of this study in non-coastal regions or among ethnically diverse populations. Therefore, further investigation through prospective randomized controlled studies is necessary to assess the broader applicability and effectiveness of the dual-dye combination with PTH test strips in diverse healthcare settings. These studies will provide a more comprehensive understanding of the intervention's potential benefits.

**Fig. 5:**
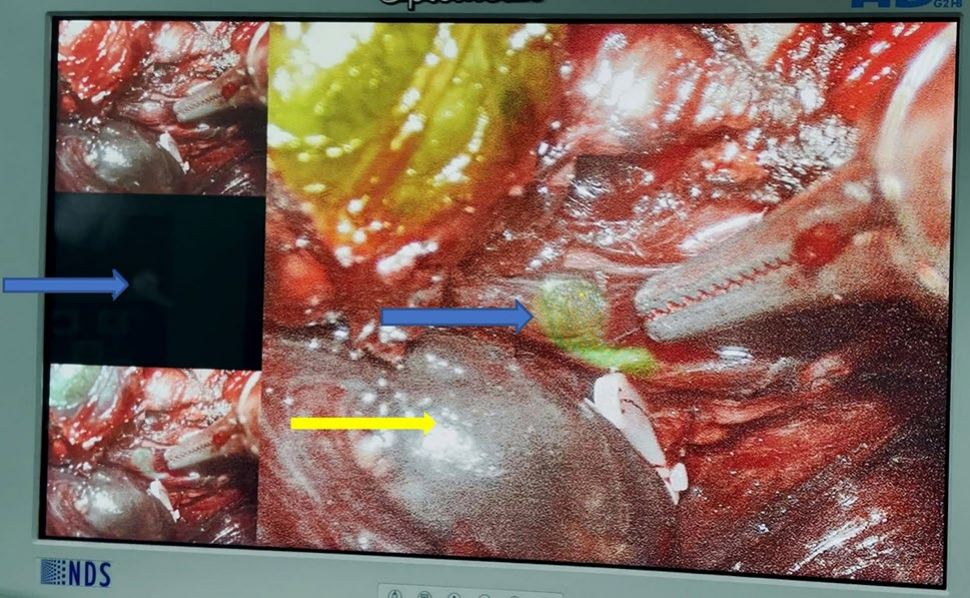
The development effect of dual dyes on parathyroid glands under fluorescence endoscopy: the positive development of ICG is depicted by the blue arrow, while the negative development of CNP is represented by the yellow arrow

The most important innovation of this study is the combined application of ICG and CNP. During the surgical procedure, we observed that the positive development of ICG was predominantly concentrated in the blood-rich vessels and glands, such as the parathyroid glands. On the other hand, the development effect of CNP is mainly in the thyroid and surrounding lymph nodes, and the negative development of the parathyroid gland is easily recognized. This combined use of dual dyes enhances the ability to distinguish and identify the parathyroid glands and their blood supply during the intraoperative period (Fig. 5). Our results showed that the double dye could offer synergistic effects and effectively reduce the interference of the imaging, allowing us to better detect the parathyroid gland and evaluate the peripheral blood supply more clearly during the operation. We also find that the ICG group had a higher rate of parathyroid autotransplantation than the CNP group, while the combined group had no statistically significant difference compared with the other two groups. This result suggests that the combined use of dual dyes can not only reduce the interference caused by the use of a single contrast agent but also provide more intraoperative evidence to inform decisions about parathyroid preservation in situ. Furthermore, we noted that the combined use of dual dye has limited contributions to the identification of the upper parathyroid gland, likely due to fact that the anatomical position of the upper parathyroid gland is relatively fixed, and the surgeon can accurately identify the upper parathyroid gland according to the standard fine dissection. None of the patients in the three groups had permanent hypoparathyroidism, which we believe may be related to the experience of the surgeons and the maturity of the current surgical techniques. In order to ensure the reliability of the experiment, we added a control group of 48 patients who underwent the same surgery but did not use dyes. A total of 2 patients developed permanent hypoparathyroidism after surgery. Our analysis found that there was no significant statistical difference among the four groups. We considered that the main reason was that the experienced surgeons on the team greatly avoided the loss of parathyroid glands during dissection. In addition, we assessed the original need during the operation. The parathyroid glands transplanted or autologously transplanted were treated promptly, which greatly ensured the normal function of the parathyroid glands (Table 4).

Another notable innovation in this study is the implementation of the highly effective PTH test paper as a criterion for determining the presence of parathyroid tissue, leading to improved accuracy in identifying parathyroid glands. It is worth highlighting that in 37 cases, both the intraoperative PTH test paper and postoperative pathology confirmed the absence of parathyroid glands. This finding underscores the reliability of the PTH test paper in accurately differentiating parathyroid tissue from non-parathyroid tissue. We speculate that using the empirical method alone may increase the false positive rate of parathyroid glands. Due to the small sample size in our study, we suggest more randomized controlled experimental studies are needed in the future to verify our hypothesis.

The analysis of the fluorescence grading scoring method reveals that patients with at least one parathyroid gland with a score of 2 had a basically normal calcium level after surgery. This effectively predict the occurrence of temporary hypoparathyroidism after surgery and help prevent the development of permanent hypoparathyroidism after surgery. This finding is consistent with the conclusions of previous studies by Vidal Fortuny et al. [16]. We recommend future research should use larger sample sizes and randomized controlled methods to further investigate the values of fluorescence grading scoring method for evaluating parathyroid activity during surgery.

Given the results from both this paper and previous studies, the combination of ICG and CNP double dye with PTH test method can significantly improve the identification number of parathyroid glands, reduce the number of false parathyroidectomy, and effectively lower the incidence of postoperative temporary hypoparathyroidism. Furthermore, the approach can reduce the interference encountered in the identification of parathyroid glands when using dyes alone. We argue that the promotion and application of this technology will require further large-scale studies to verify its efficacy. This will facilitate the adoption of safer and more efficient PTC surgeries in hospitals at all levels, reduce parathyroid related complications, and enhance the overall quality of surgical procedures.

### Supplementary Information

Below is the link to the electronic supplementary material.

Supplementary file1 (XLSX 59 KB)

## Data Availability

The datasets generated during and/or analyzed during the current study are available from the corresponding author, Jiagen Li, on reasonable request.
